# White matter microstructure in mid- to late adulthood is influenced by pathway-stratified polygenic risk for Alzheimer’s disease

**DOI:** 10.3389/fnins.2025.1638503

**Published:** 2025-10-28

**Authors:** Judith R. Harrison, Sonya F. Foley, Emily Simmonds, Matthew Bracher-Smith, Peter Holmans, Evie Stergiakouli, Xavier Caseras, Valentina Escott-Price, Derek K. Jones

**Affiliations:** ^1^Institute of Neuroscience, Biomedical Research Building, Campus for Ageing and Vitality, Newcastle University, Newcastle upon Tyne, United Kingdom; ^2^Cardiff University Brain Research Imaging Centre (CUBRIC), Cardiff University, Cardiff, United Kingdom; ^3^UK Dementia Research Institute, Division of Neuroscience and Mental Health, School of Medicine, Cardiff University, Cardiff, United Kingdom; ^4^Division of Psychological Medicine and Clinical Neurosciences, School of Medicine, Cardiff University, Cardiff, United Kingdom; ^5^Bristol Medical School, University of Bristol, Bristol, United Kingdom; ^6^MRC Integrative Epidemiology Unit at the University of Bristol, Bristol, United Kingdom; ^7^Mary MacKillop Institute for Health Research, Australian Catholic University, Melbourne, VIC, Australia

**Keywords:** Alzheimer’s disease, genetic predisposition to disease, genome-wide association study, diffusion magnetic resonance imaging, white matter, lipid metabolism, tau proteins, ALSPAC

## Abstract

**Introduction:**

Alzheimer’s disease involves progressive white matter microstructural degeneration that may precede clinical symptoms by decades. While polygenic risk scores (PRS) quantify cumulative genetic liability for AD, genome-wide PRS lack mechanistic specificity. We tested whether pathway-specific PRS, targeting areas of biology including tau binding, lipid metabolism, and immune response, are differentially associated with diffusion MRI measures across the lifespan.

**Methods:**

We analyzed two population-based cohorts: the Avon Longitudinal Study of Parents and Children (ALSPAC; mean age = 19.8 years, *n* = 517) and UK Biobank (mean age = 64.2 years, *n* = 18,172). Genome-wide and nine pathway-specific PRS for Alzheimer’s disease were constructed using GWAS summary statistics and a clumping threshold of r^2^ < 0.2 at *p* < 0.001. Diffusion MRI data were processed separately within each cohort: in ALSPAC, tract-based fractional anisotropy (FA) and mean diffusivity (MD) were extracted using probabilistic tractography from native-space regions of interest; in UK Biobank, diffusion metrics were derived from TBSS-aligned skeletons and standard atlas-based ROIs. Analyses focused on three tracts vulnerable to early AD pathology: the dorsal cingulum, parahippocampal cingulum, and fornix. Multiple linear regression models were used to assess PRS associations with FA and MD, adjusting for demographic, scanner, and genetic ancestry covariates. False discovery rate correction addressed multiple comparisons, and sensitivity analyses were performed excluding the APOE region.

**Results:**

In UK Biobank, higher PRS for protein–lipid complex assembly and tau protein binding were robustly associated with lower fractional anisotropy and higher mean diffusivity in both dorsal and parahippocampal cingulum segments (False discovery rate-corrected *p* < 0.05), explaining more variance than *APOE* alone; no significant effects emerged in the fornix. Genome-wide PRS showed weaker, non-significant associations. In ALSPAC, no PRS metric survived FDR correction, though nominal trends appeared in the dorsal cingulum. Sensitivity analyses confirmed that key cingulum associations in older adults persisted after omitting *APOE*.

**Conclusion:**

Pathway-specific polygenic risk for Alzheimer’s disease manifests in white matter microstructure by mid- to late adulthood but not in early adulthood, suggesting an age-dependent emergence of genetic effects. dMRI phenotypes may thus serve as intermediate biomarkers for dissecting mechanistic pathways of preclinical Alzheimer’s disease vulnerability.

## Introduction

Alzheimer’s disease (AD) is a progressive neurodegenerative condition that represents a major global health challenge, with prevalence rates estimated between 5% and 7% in adults over 60 years of age (Prince et al., 2013). While a small subset of cases result from rare autosomal dominant mutations in genes such as *APP*, *PSEN1*, and *PSEN2* (Tanzi, 2012), most are due to the interplay of complex genetic and environmental factors. Genome-wide association studies (GWAS) have identified close to 80 loci associated with AD risk, with the *APOE* ε4 allele representing the most significant contributor (Marioni et al., 2017; Jansen et al., 2019; Kunkle et al., 2019; Bellenguez et al., 2022). Polygenic risk scores (PRS), which aggregate the genetic risk across these loci, offer a powerful approach to quantify the cumulative genetic burden for AD (Escott-Price et al., 2015; Harrison et al., 2020b). PRS have been linked to structural brain changes, including cortical thinning and subcortical atrophy, which are established markers of AD-related pathology (Mak et al., 2017; Harrison et al., 2023). However, studies focusing on PRS and white matter microstructure, which is an important mediator of brain network integrity, remain limited.

White matter signal changes, such as reduced fractional anisotropy (FA) and increased mean diffusivity (MD), are putative indicators of microstructural degeneration and have been reported in both symptomatic and preclinical stages of AD (Kantarci et al., 2014; Alm and Bakker, 2019). These changes may emerge decades before cognitive symptoms, particularly in key tracts such as the parahippocampal cingulum and fornix, which are vulnerable to early AD pathology (Zhuang et al., 2013; Wen et al., 2019). Combining PRS with diffusion MRI (dMRI)-derived metrics offers a promising avenue for detecting early, preclinical indicators of AD risk. Yet, most AD PRS studies have concentrated on cortical or subcortical volumes (Mak et al., 2017), with few investigations of white matter pathways (Harrison et al., 2020a).

In addition to understanding overall genetic risk, pathway-specific PRS provide a more granular approach by quantifying genetic burden within defined biological pathways, such as those related to amyloid processing, tau binding, and immune response (Kunkle et al., 2019; Vogrinc et al., 2021). This could enable researchers to delineate the mechanistic links between genetic risk and neuroimaging phenotypes. Emerging evidence suggests that pathway-specific PRS may explain more variance in brain structure than genome-wide PRS, particularly in cortical regions and subcortical volumes implicated in AD (Ahmad et al., 2018; Caspers et al., 2020; Harrison et al., 2023). Recent work has linked AD polygenic risk to white-matter alterations in UK Biobank (Lorenz et al., 2025); our contribution is to interrogate pathway-specific PRS and their age-dependent expression.

To address this gap, we tested associations between nine biologically informed AD PRS and diffusion MRI measures (FA, MD) in two population cohorts: we compared a young adult cohort (ALSPAC; ∼20 years) with an older adult cohort (UK Biobank; ∼64 years) to test a lifespan hypothesis of age-dependent genetic expression. By directly comparing these age groups, we evaluate whether pathway-specific genetic risk manifests in white matter early in adulthood, and how those effects might evolve with age.

### Hypotheses

1. Higher pathway-specific PRS will be linked to lower FA and higher MD in AD-vulnerable tracts.
2. Associations will be stronger in older versus younger adults, reflecting accumulated effects of genetic liability on white matter structure.

## Materials and methods

### Participants

Participants were drawn from two population-based cohorts: the Avon Longitudinal Study of Parents and Children (ALSPAC) (Boyd et al., 2013) and UK Biobank (Sudlow et al., 2015). For ALSPAC, pregnant women resident in Avon, UK with expected dates of delivery between 1st April 1991 and 31st December 1992 were invited to take part in the study. The initial number of pregnancies enrolled was 14,541; 13,988 children were alive at 1 year of age. Additional enrollments brought the total sample size for analyses using any data collected after the age of seven to 15,447 pregnancies, with 14,901 children were alive at 1 year of age. The ALSPAC cohort analyzed in the present study comprised younger adults recruited for neuroimaging studies at approximately 19 years of age. Ethical approval for the study was obtained from the ALSPAC Ethics and Law Committee and the Local Research Ethics Committees (Boyd et al., 2013; Northstone et al., 2019). Following genotyping and imaging quality control, 517 participants (80.7% male; mean age: 19.81 years, SD: 0.02) with high-quality structural T1 and diffusion MRI data were included (Sharp et al., 2020).

UK Biobank is an ongoing longitudinal cohort study of over 500,000 participants. A subset of 100,000 individuals is being recalled for multimodal imaging, and the first 20,000 datasets were analyzed in this study (Sudlow et al., 2015). Ethical approval for UK Biobank was granted by several organizations (UK Biobank, 2007). UK Biobank data were accessed under application number 17044. All analyses reported here were conducted using the 2023 data release and completed during the active approval period of the project. Data use was in full compliance with the UK Biobank Material Transfer Agreement and data access conditions in place at the time. UK Biobank participants were excluded if they self-reported a history of neurological or major psychiatric disorders at baseline or follow-up, or if hospital admission records indicated conditions such as substance abuse/dependency, bipolar disorder, schizophrenia/psychosis, neurodegenerative disorders, dementia, or intellectual disability. After quality control, 18,172 individuals (47.3% male; mean age: 64.2 years, SD: 7.75) with diffusion MRI and genetic data were included.

Participants from both cohorts were excluded if they did not report white British or Irish descent, or if they requested data withdrawal. In UK Biobank, this ancestry was defined using the “white British ancestry subset” field, which combines self-reported ethnicity with principal component-based genetic clustering, to ensure population homogeneity for PRS calculation. The study adhered to the principles of the Human Tissue Act (2004), and all participants provided written informed consent. Further details of participant recruitment and exclusion criteria are described in previously published work (Harrison et al., 2023).

### Genotyping

Genotyping data for ALSPAC participants were obtained using the Illumina HumanHap550 quad SNP genotyping platform (Illumina Inc., San Diego, CA, USA), while UK Biobank data were derived from the Affymetrix UK BiLEVE Axiom and UK Biobank Axiom arrays.^1^ Quality control was conducted using PLINK, with exclusions applied for genotyping completeness below 97%, minor allele frequency (MAF) less than 1%, and deviations from Hardy-Weinberg equilibrium (*p* < 1 × 10^–4^) (Purcell et al., 2007). Genotype imputation was carried out using a prephasing and imputation strategy implemented in IMPUTE2 and SHAPEIT (Howie et al., 2011; Delaneau et al., 2012), using the 1000 Genomes Project Phase I integrated variant set (December 2013 release) as the reference panel (1000 Genomes Project Consortium et al., 2015).

### Polygenic risk score (PRS) calculations

As described previously (Harrison et al., 2023), PRS were calculated using GWAS summary statistics from the largest clinically-defined AD study available (Kunkle et al., 2019), that does not include either of the ALSPAC or UK Biobank cohorts. SNPs with a minor allele frequency below 1% were excluded from analyses. To account for linkage disequilibrium (LD), the data were pruned using the clumping procedure in PLINK, with a threshold of r^2^ > 0.2 and a 500 kb window (–clump-r2 and –clump-kb parameters). Polygenic risk scores (PRS) were then calculated using the PLINK –score function (Purcell et al., 2007). Based on prior research demonstrating that a *p*-value threshold (PT) of 0.001 captures the greatest variance in brain structural phenotypes linked to AD risk (Foley et al., 2016), this threshold was used for the primary analysis. Secondary analyses evaluated a range of *p*-value thresholds spanning more and less stringent settings relative to PT = 0.001 (0.5, 0.3, 0.1, 0.01, 1 × 10^–4^, 1 × 10^–5^, 1 × 10^–6^).

To derive pathway-specific PRS, we used the set of disease-relevant biological pathways identified by Kunkle et al. (2019), who reported nine Gene Ontology (GO) terms significantly enriched for AD-associated variants using the MAGMA gene-set analysis tool (de Leeuw et al., 2015). These pathways include: protein–lipid complex assembly; regulation of Aβ formation; protein–lipid complex; regulation of amyloid precursor protein catabolic process; tau protein binding; reverse cholesterol transport; protein–lipid complex subunit organization; plasma lipoprotein particle assembly; and activation of immune response. Further methodological details on the MAGMA pathway analysis, including the n genes and n SNPs in UK Biobank and ALSPAC pathways, can be found in our previous publication (Harrison et al., 2023). Genes within each pathway were used to generate pathway-specific SNP sets, which were then aligned with the discovery GWAS summary statistics. These pathway PRS were computed following the same clumping and scoring procedure used for the genome-wide PRS.

### MRI data acquisition

For the ALSPAC cohort, MRI data were acquired at Cardiff University Brain Research Imaging Center (CUBRIC) using a 3T General Electric HDx scanner and an 8-channel head coil. T1-weighted structural images were collected using a 3D fast spoiled gradient echo (FSPGR) sequence with 168–182 oblique-axial AC-PC slices, 1 mm isotropic resolution, flip angle = 20°, TR/TE/TI = 7.9 or 7.8/3.0/450 ms, slice thickness = 1 mm, field of view = 256 mm × 192 mm, and acquisition time between 6 and 10 min (Sharp et al., 2020). Diffusion MRI data were acquired using a pulsed gradient spin echo EPI sequence with 60 diffusion directions at *b* = 1,200 s/mm^2^, 5 b = 0 s/mm^2^ volumes, voxel size = 2.4 mm isotropic, TR = 16.5 s, TE = 87 ms, and a total scan duration of approximately 13 min.

For the UK Biobank cohort, MRI data were obtained across three dedicated imaging centers using Siemens Skyra 3T scanners and standard Siemens 32-channel head coils. T1-weighted structural images were acquired using a 3D MPRAGE sequence with sagittal orientation, TR = 2,000 ms, TI = 880 ms, voxel size = 1 mm^3^ isotropic, matrix = 208 mm × 256 mm × 256 mm, and scan time of approximately 5 min (Alfaro-Almagro et al., 2018). Diffusion MRI was conducted with a multi-shell acquisition comprising 100 diffusion directions at *b*-values of 1,000 and 2,000 s/mm^2^, along with 6 b = 0 s/mm^2^ volumes, voxel size = 2 mm^3^ isotropic, TR = 3.6 s, TE = 92 ms, and a scan duration of approximately 7 min.

### Diffusion MRI data processing

**FIGURE 1:**
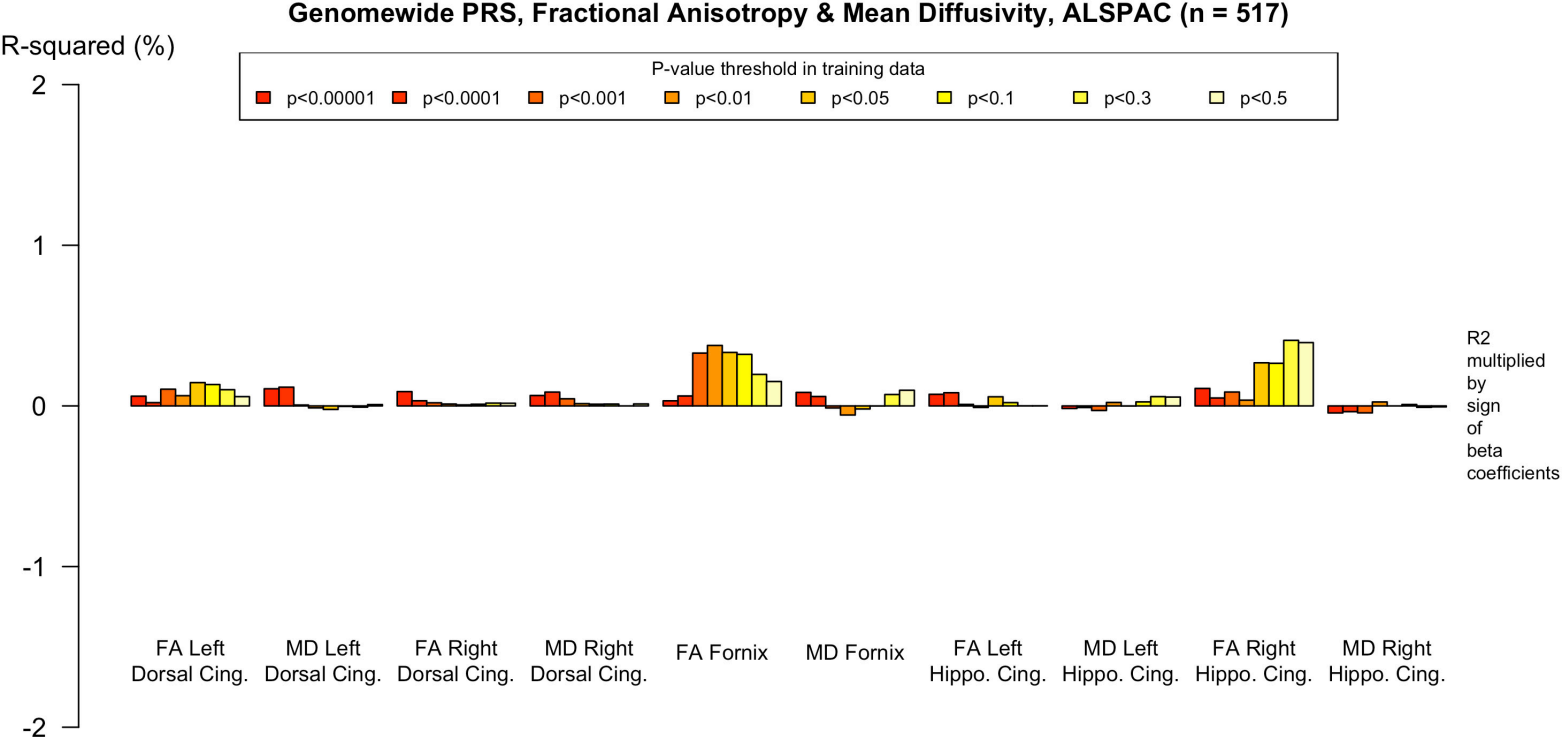
Example dMRI regions of interest defined for ALSPAC. Left image: the fornix; Center image: the dorsal cingulum; Right image: the parahippocampal cingulum.

For the ALSPAC cohort, diffusion MRI data were processed using tools from FSL (Smith et al., 2004) and MRtrix3 (Tournier et al., 2012). Raw diffusion-weighted images underwent correction for eddy current-induced distortions and participant motion using FSL’s *eddy*. Non-brain tissue was removed using the Brain Extraction Tool (BET), and diffusion tensors were fitted at each voxel using *dtifit* to generate FA and MD maps. These maps were non-linearly registered to MNI152 standard space. Regions of interest (ROIs), including the parahippocampal cingulum, dorsal cingulum and fornix, were delineated using probabilistic tractography based on anatomical priors (see examples, Figure 1). Mean FA and MD values were extracted from these tracts for downstream statistical analysis.

In the UK Biobank cohort, diffusion data were preprocessed using the standardized UK Biobank pipeline (Alfaro-Almagro et al., 2018). This included correction for eddy currents, head motion, and gradient distortion. Diffusion tensor imaging metrics were derived from the *b* = 1,000 s/mm^2^ shell using FSL’s *dtifit*. The resulting FA images were aligned to MNI space and processed with tract-based spatial statistics (TBSS) to project individual data onto a mean white matter skeleton (Smith et al., 2006). Mean FA and MD values were extracted from predefined white matter ROIs based on the JHU-ICBM tractography atlas for comparative analysis across participants.

### Statistical analysis

Statistical analyses were conducted using R v3.6.3 (R Core Team, 2020). Modeling used base stats:lm and related functions; figures were generated with “ggplot2.” Polygenic risk scores were z-standardized prior to analysis. FA and MD values were used in their original units, as derived from diffusion MRI processing pipelines. Multiple linear regression models were used to assess the association between PRS and FA/MD in anatomically defined tracts. Separate models were constructed for genome-wide PRS and each of the nine pathway-specific PRS. For each tract and hemisphere, diffusion metrics (FA or MD) were the dependent variables. Separate linear models were fit for the genome-wide PRS and each pathway-specific PRS, with the PRS as the predictor of interest and the following covariates: age, sex, intracranial volume; UK Biobank models additionally included imaging center (site) and genotyping array; ancestry was controlled via principal components (10 for ALSPAC; 15 for UK Biobank, in accordance with cohort recommendations; (Fraser et al., 2013; Sudlow et al., 2015). Genotyping array was included for UK Biobank to account for platform/imputation batch effects that can influence PRS values.

To account for multiple comparisons across imaging phenotypes and PRS models, *p*-values were corrected using the False Discovery Rate (FDR) procedure (Benjamini et al., 2001). Secondary analyses included re-estimation of all models excluding SNPs within the *APOE* genomic region (chr19:44.4 Mb–46.5 Mb) to determine *APOE*-independent effects. Additional analyses were conducted using *APOE*-only PRS to evaluate its relative explanatory power. Finally, associations were examined across a range of *p*-value thresholds (PT = 0.5, 0.3, 0.1, 0.01, 1 × 10^–4^, 1 × 10^–5^, 1 × 10^–6^) to assess the consistency of effects under varying inclusion criteria for SNPs.

## Results

### Associations between AD PRS and white matter microstructure in older adults

In the older adults, multiple pathway-specific PRS were significantly associated with white matter microstructure measures, particularly in the parahippocampal cingulum and dorsal cingulum. These results are summarized in Supplementary Tables 1, 2. Associations that withstood correction for multiple comparisons and those that explained more variance than *APOE* are indicated.

Patterns of association were consistent for most pathway PRS. For example, the protein–lipid complex assembly pathway PRS was negatively associated with FA in the parahippocampal cingulum, on the left (*p* = 0.001; Beta = −8.43 × 10^–4^; 95% CI −1.36 × 10^–3^, −3.28 × 10^–4^; r^2^ = 5.8 × 10^–4^) and on the right (*p* = 3.89 × 10^5^; Beta = −1.09 × 10^–3^; 95% CI −1.62 × 10^–3^, −5.73 × 10^–4^; r^2^ = 9.4 × 10^–4^). However, there were no associations with FA in the dorsal cingulum. For MD, the protein–lipid complex assembly pathway PRS was positively associated in the left and right parahippocampal cingulum (*p* = 0.005; Beta = 7.58 × 10^–7^; 95% CI 2.33 × 10^–7^, 1.28 × 10^6^; r^2^ = 4.7 × 10^–4^ and *p* = 0.005; Beta = 8.19 × 10^–7^; 95% CI 3.0 × 10^–7^, 1.34 × 10^6^; r^2^ = 5.7 × 10^–4^, respectively). There was also a positive association with MD in the left and right dorsal cingulum (*p* = 1.64 × 10^–4^; Beta = 8.4 × 10^–7^; 95% CI 4.03 × 10^–7^, 1.28 × 10^6^; r^2^ = 1.6 × 10^–4^ and *p* = 3.94 × 10^–4^; Beta = 8.04 × 10^–7^; 95% CI 3.59 × 10^–7^, 1.25 × 10^6^; r^2^ = 3.9 × 10^–4^, respectively). The only pathway PRS with a different pattern of association was the immune response PRS, with no significant effects.

The genome-wide PRS showed less evidence of association with white matter microstructure than the pathway PRS. There were nominally significant associations with reduced FA in the right parahippocampal cingulum and increased MD in the left dorsal cingulum, but these did not withstand FDR correction, as indicated in the Supplementary Tables.

### Associations between AD PRS and white matter microstructure in younger adults

In the younger adult cohort, there was less evidence of association between PRS and white matter microstructure. The direction of the effect seen inconsistent across ROIs and PRS, and r^2^ indicted minimal variance was explained (r^2^ up to 5.5 × 10^–7^). Two nominally significant associations were observed. For instance, there was evidence of a positive association between MD in the left dorsal cingulum and the regulation of amyloid precursor protein catabolic process pathway PRS (*p* = 0.042; Beta = 2.21 × 10^6^; 95% CI 2.21 × 10^8^, 4.34 × 10^6^; r^2^ = 7.84 × 10^–3^) and the protein−lipid complex subunit organization PRS both showed (*p* = 0.043, Beta = 2.26 × 10^6^; 95% CI 6.94 × 10^8^, 4.26 × 10^6^; r^2^ = 7.9 × 10^–3^). See Supplementary Tables 3, 4 for a summary of results.

### *APOE*-independent and *APOE*-specific effects

Several of the significant associations observed in the UK Biobank cohort showed corrected significance after excluding the *APOE* region from the PRS, indicating that these effects were not solely driven by *APOE*-related variants. Associations which remained significant when *APOE* was removed from the PRS are indicated in Supplementary Tables 1–4. For example, the tau protein binding pathway PRS was negatively associated with FA in the left and right parahippocampal cingulum (*p* = 0.001; Beta = −8.43 × 10^–4^; 95% CI −1.36 × 10^–3^, −3.28 × 10^–4^ and *p* = 3.91, Beta = −1.09 × 10^–3^; 95% CI −1.61 × 10^–3^, −5.73 × 10^–4^, respectively), and this effect was still significant (with FDR correction) when *APOE* was excluded (see Supplementary Figure 1). Similarly, there were also significant positive associations with the tau pathway PRS and MD in the dorsal cingulum and parahippocampal cingulum bilaterally which persisted without *APOE* (p range = 0.005–1.64 × 10^–4^, see Supplementary Tables 1–4). In contrast, in the ALSPAC younger adult cohort, the exclusion of *APOE* led to attenuation of all previously nominal associations and reduced effect sizes. Analyses using an *APOE*-only PRS showed that although *APOE* evidently contributed to white matter variation in UK Biobank, in several cases, pathway-specific scores explained a greater proportion of the variance in white matter microstructure than *APOE* alone (see Supplementary Tables 1–4).

### PRS threshold sensitivity analyses

**FIGURE 2:**
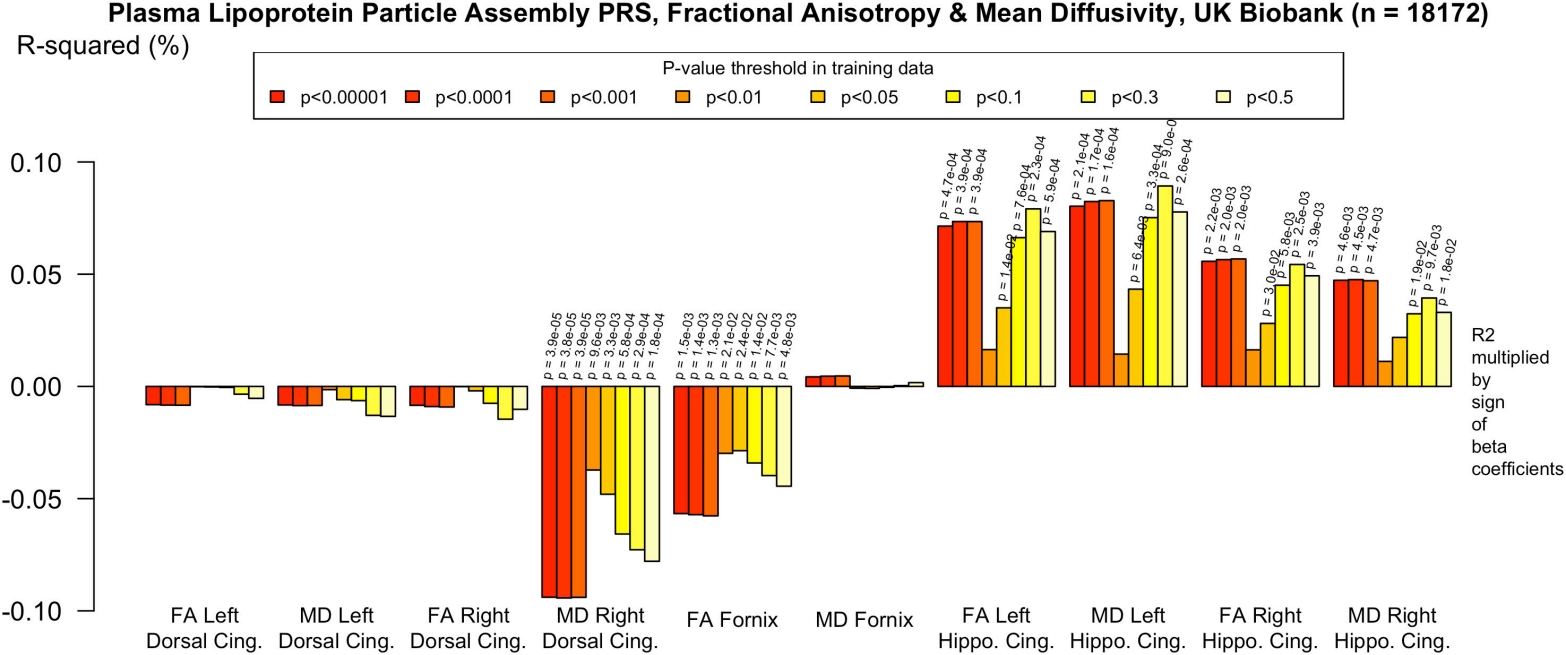
Associations with the protein-lipid complex assembly PRS and diffusion metrics in UK Biobank (*n* = 18 172). FA/MD are outcomes and PRS are predictors. Pathway-specific polygenic scores were negatively associated with FA in the dorsal and parahippocampal cingulum and positively associated with MD in the same regions. There were no positive associations with FA or MD in the fornix. Imaging phenotypes are shown on the *x*-axis, the R^2^ multiplied with the sign of the B-coefficients (positive and negative) are shown on the *y*-axis. Any nominally significant results are labeled with their nominal *P*-value. Each bar represents a version of the PRS, color-coded by the *P*-value threshold used in the training data, shown on the legend. “*P*-value threshold” denotes the SNP inclusion threshold for PRS construction (not a training/validation split). Numerical coefficients, standard errors, confidence intervals, and *p*-values for each model are provided in Supplementary Tables. PRS, polygenic risk score; FA, fractional anisotropy; MD, mean diffusivity; UKBB, UK Biobank; ROI, Region of Interest; SNP, Single Nucleotide Polymorphism; R^2^, Coefficient of Determination; B, Regression Coefficient.

**FIGURE 3:**
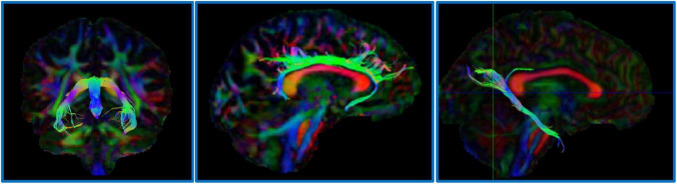
Associations with the genome-wide PRS and diffusion metrics in UK Biobank (*n* = 18 172). FA/MD are outcomes and PRS are predictors. Genome-wide polygenic score was not significantly associated with any white matter metrics at the PT in our primary analysis (PT 0.001). There were trends toward associations at more liberal PT, however none withstood multiple comparisons correction and the direction of effect was the reverse of what would be expected in some cases, e.g., decreased MD in the right dorsal cingulum and increased FA in the left parahippocampal cingulum. Imaging phenotypes are shown on the *x*-axis, the R^2^ multiplied with the sign of the B-coefficients (positive and negative) are shown on the *y*-axis. Any nominally significant results are labeled with their nominal *P*-value. Each bar represents a version of the PRS, color-coded by the *P*-value threshold used in the training data, shown on the legend. “*P*-value threshold” denotes the SNP inclusion threshold for PRS construction (not a training/validation split). Numerical coefficients, standard errors, confidence intervals, and *p*-values for each model are provided in Supplementary Tables. PRS, polygenic risk score; FA, fractional anisotropy; MD, mean diffusivity; UKBB, UK Biobank; ROI, Region of Interest; SNP, Single Nucleotide Polymorphism; R^2^, Coefficient of Determination; B, Regression Coefficient.

**FIGURE 4:**
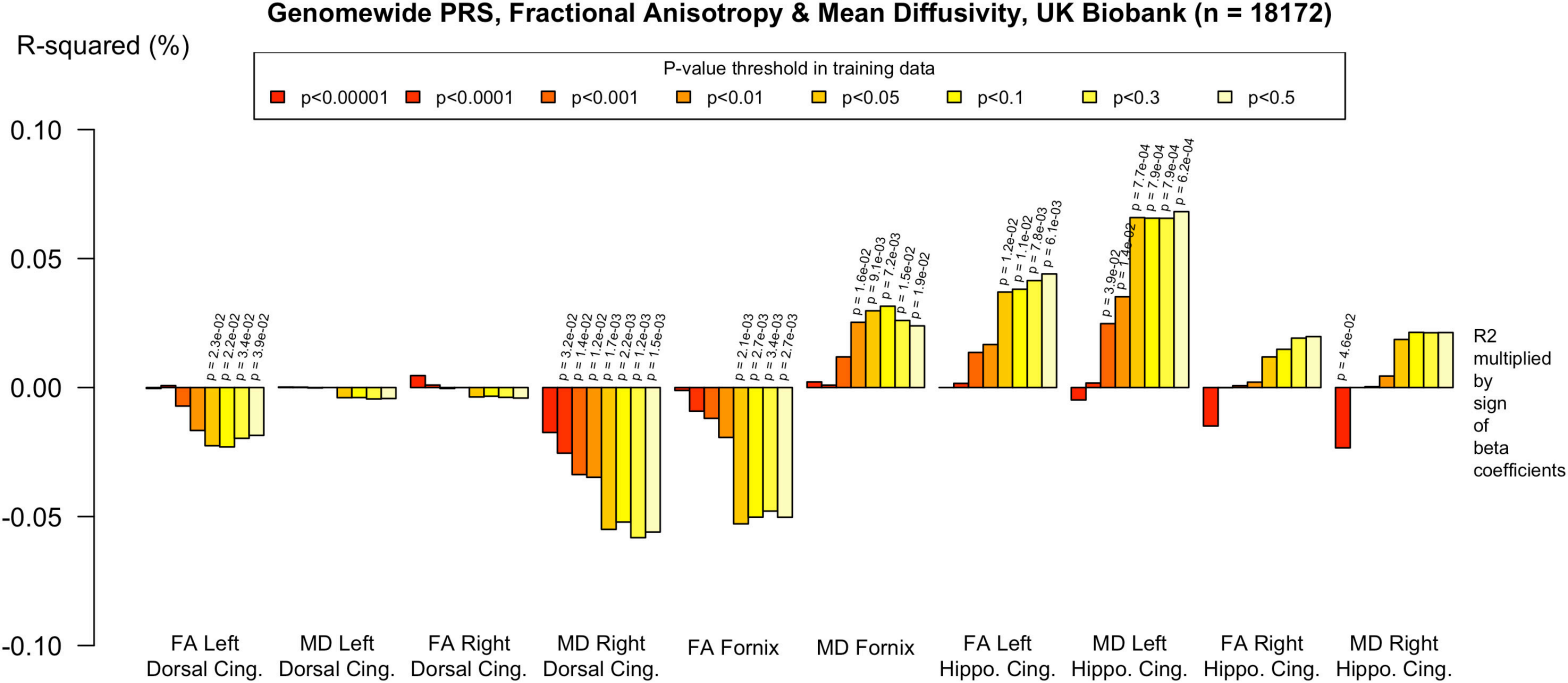
Associations with the protein-lipid complex assembly PRS and diffusion metrics in ALSPAC (*n* = 517). FA/MD are outcomes and PRS are predictors. There were no associations that survived multiple comparisons correction even at more liberal PTs. There was a trend toward association with increased MD in the left dorsal cingulum. Imaging phenotypes are shown on the *X* axis, the R^2^ multiplied with the sign of the B-coefficients (positive and negative) are shown on the *Y* axis. Any nominally significant results are labeled with their nominal *p*-value. Each bar represents a version of the PRS, color-coded by the *p*-value threshold used in the training data, shown on the legend. “*P*-value threshold” denotes the SNP inclusion threshold for PRS construction (not a training/validation split). Numerical coefficients, standard errors, confidence intervals, and *p*-values for each model are provided in Supplementary Tables. PRS, polygenic risk score; FA, fractional anisotropy; MD, mean diffusivity; UKBB, UK Biobank; ROI, Region of Interest; SNP, Single Nucleotide Polymorphism; R^2^, Coefficient of Determination; B, Regression Coefficient.

**FIGURE 5:**
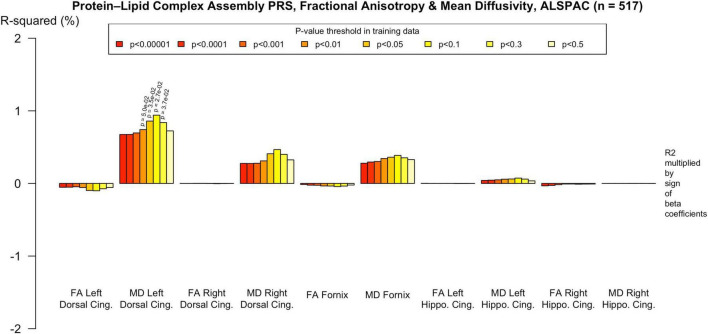
Associations with the genome-wide PRS and diffusion metrics in ALSPAC (*n* = 517). FA/MD are outcomes and PRS are predictors. There were no associations between any PT and FA or MD. Imaging phenotypes are shown on the *X* axis, the R^2^ multiplied with the sign of the B-coefficients (positive and negative) are shown on the *Y* axis. Any nominally significant results are labeled with their nominal *p*-value. Each bar represents a version of the PRS, color-coded by the *p*-value threshold used in the training data, shown on the legend. “*P*-value threshold” denotes the SNP inclusion threshold for PRS construction (not a training/validation split). Numerical coefficients, standard errors, confidence intervals, and *p*-values for each model are provided in Supplementary Tables. PRS, polygenic risk score; FA, fractional anisotropy; MD, mean diffusivity; UKBB, UK Biobank; ROI, Region of Interest; SNP, Single Nucleotide Polymorphism; R^2^, Coefficient of Determination; B, Regression Coefficient.

To assess the robustness of associations across varying degrees of SNP inclusion, analyses were repeated using a range of additional *p*-value thresholds (PTs) for PRS construction: 0.5, 0.3, 0.1, 0.01, 1 × 104, 1 × 105, and 1 × 106. These are shown on Figures 2–5. In the UK Biobank cohort, significant associations between white matter microstructure and pathway-specific PRS were most consistently observed at PT = 0.001 and other more stringent thresholds, particularly for the tau protein binding and protein–lipid complex assembly pathways (shown in Figures 2–5, Supplementary Figure 1). The genome-wide PRS showed some trends toward significance at more liberal thresholds, however they didn’t remain when corrected for multiple comparisons and the direction of effect was often reversed. For example, there was an apparent positive association with FA in the left hippocampal cingulum at PTs > 0.05. In contrast, in the ALSPAC cohort, none of the associations reached significance at any threshold following correction for multiple comparisons, although nominal effects occasionally varied by PT. Overall, these findings support PT = 0.001 as the optimal threshold for capturing variance in white matter microstructure associated with AD genetic risk, consistent with prior literature (Foley et al., 2016).

## Discussion

This study provides evidence that AD-related genetic risk, when partitioned by biological pathway, is associated with variation in white matter microstructure in mid-to-late adulthood, but not in early adulthood. Using large population cohorts at two developmental stages, we identified tract-specific associations between higher pathway-specific polygenic scores and diffusion MRI markers of white matter microstructure in older adults, with no significant findings in younger adults after correction for multiple comparisons. These findings add to emerging evidence that the influence of AD genetic liability on brain structure may be age-dependent (Jiaxuan Peng et al., 2024; Korologou-Linden et al., 2025), with expression of risk increasing with advancing age.

In the UK Biobank cohort of older adults, pathway-specific PRS, particularly those linked to tau protein binding, lipid and amyloid metabolism, were significantly associated with increased MD and decreased FA in the parahippocampal cingulum and dorsal cingulum. The strongest associations were observed for MD in these regions, with more modest negative correlations between PRS and FA in the parahippocampal cingulum. These effects were significant even after exclusion of the *APOE* region and were stronger than those observed with an *APOE* alone. No associations were identified with either MD or FA in the fornix at the primary *p*-value threshold (PT = 0.001), although some emerged in secondary analysis of more liberal thresholds. The genome-wide PRS showed weaker evidence overall, with no associations surviving correction.

In the ALSPAC cohort of younger adults, no associations between AD-related genetic risk and white matter microstructure survived correction for multiple comparisons. Nonetheless, nominal associations were observed, including between higher PRS and increased MD in the left cingulum, although these effects were small and not statistically robust. However, several nominal associations showed the opposite direction of effect compared to older adults, raising the possibility of age-dependent modulation or developmental non-linearity in the expression of AD genetic risk (Lopez et al., 2012; Bonham et al., 2016). Given differences in acquisition and processing pipelines between cohorts, diffusion metrics were not standardized within samples, and thus direct comparisons of beta coefficients should be interpreted cautiously. However, r^2^ values, which are scale independent, were generally smaller in ALSPAC than UK Biobank, suggesting that the lack of significant associations is not solely attributable to sample size, but also reflects weaker underlying effects. White matter microstructure continues to mature throughout adolescence and early adulthood (Paus, 2010; Tamnes et al., 2010), and previous studies have suggested that age-related neurodevelopmental changes may mask or modulate the influence of genetic risk variants during this period (Giedd et al., 1999; Smith et al., 2016). Indeed, studies have demonstrated changes in white matter microstructure in young *APOE* or clusterin risk allele carriers (Braskie et al., 2011; Heise et al., 2011), and in infants carrying *APOE*4, with altered myelin development detectable within the first year of life (Dean et al., 2014; Remer et al., 2020). These findings support the notion that while AD risk variants may influence white matter structure early in life, the phenotypic effects may remain subtle or regionally specific until later stages of development or aging.

The link between changes in white matter signal and poorer cognitive function has been demonstrated across several neurodegenerative cohorts, further highlighting white matter metrics as promising markers for preclinical detection of AD vulnerability (Acosta-Cabronero et al., 2012; Power et al., 2019). The absence of corrected associations in ALSPAC, contrasted with robust effects in UK Biobank, supports a developmental timing model in which pathway-specific AD liability becomes phenotypically expressed in mid- to late adulthood. These patterns are consistent with recent evidence showing that the influence of AD-related genetic risk on brain structure may be latent in early life and become phenotypically expressed through age-accelerated neurodegeneration in mid-to-late adulthood (Jiaxuan Peng et al., 2024; Korologou-Linden et al., 2025). Indeed, developmental mismatch models suggest that genetically vulnerable white matter circuits may follow altered maturational trajectories, potentially laying a structural foundation for later neurodegenerative processes (Mills et al., 2014).

To our knowledge, this is the first study to investigate white matter microstructure in relation to pathway-specific AD PRS. Prior research has focused on gray matter phenotypes. Three previous studies applied pathway-specific PRS in dementia-free older adult cohorts but only used only used Bonferroni significant loci from GWAS. Corlier et al. (2018) (*N* = 355) found that an immune response PRS (comprising 11 SNPs) was associated with a global measure of cortical thinning. Ahmad et al. (2018) (*N* = 4,521) reported no significant associations between seven pathway-based PRS (each with ∼20 SNPs) and hippocampal or whole brain volumes. Caspers et al. (2020) (*N* = 544) identified associations between pathway-specific PRS and cortical thinning, noting more bilateral effects and distinct patterns involving superior parietal and anterior/mid-cingulate regions. Our previous work using UK Biobank and ALSPAC data showed no significant associations between AD PRS and gray matter volumes in younger adults (Harrison et al., 2023), consistent with the null white matter results in the present study.

A growing body of evidence suggests that the neuroanatomical effects of Alzheimer’s disease genetic risk are developmentally regulated, with expression emerging gradually across the lifespan. He et al. (2023) and Korologou-Linden et al. (2025) demonstrated age-dependent PRS effects on brain morphology across large datasets (*N* > 20,000), with associations absent in youth but prominent in mid-to-late adulthood (p. 20). Similarly, Jiaxuan Peng et al. (2024) reported that higher AD PRS was linked to reduced white matter signal changes and network efficiency in older cohorts, particularly in tracts implicated in AD progression. Network-based approaches may be more sensitive to subtle white matter changes in young adults. For example, Mirza-Davies et al. (2022) used diffusion MRI-derived connectome analyses in the ALSPAC cohort and found that higher genome-wide AD PRS was associated with reduced connectivity in visual and rich-club brain regions. Our findings extend this evidence by showing that pathway-specific scores track with microstructural disruption in these same regions, and that several effects remain after removing *APOE*, underscoring the value of polygenic approaches that move beyond single-gene models (Escott-Price et al., 2015).

This study has several notable strengths. First, it is the largest to date to examine white matter microstructure in relation to pathway-specific polygenic risk for Alzheimer’s disease, using harmonized genetic pipelines across two well-characterized population cohorts at different life stages. Second, we applied summary statistics from the largest GWAS of clinically-defined AD (Kunkle et al., 2019), and were able to construct threshold-based PRS with increased statistical power and more comprehensive genetic signal compared to previous studies that relied solely on genome-wide significant loci. The large sample sizes in both cohorts provided sufficient power to detect subtle associations, while the use of biologically informed pathway scores allowed for a more mechanistically nuanced investigation of AD risk architecture.

Several limitations must be acknowledged. The ALSPAC cohort was much smaller than the UKBB cohort, and therefore may not have been powered to detect very subtle effects. Although the same quality control procedures and PRS construction pipeline were applied across both cohorts, minor differences in SNP availability may have resulted in variation in the SNPs retained after LD clumping, potentially affecting the comparability of the resulting scores. We used 1000 Genomes phase I imputation, which is robust for common variants but may underperform TOPMed for lower-frequency alleles; this could modestly reduce PRS fidelity. Incorporating TOPMed-based imputation in future studies may enhance sensitivity. Both ALSPAC and UK Biobank reflect relatively healthy, high-functioning populations, which may limit generalizability, and the ALSPAC imaging subsample was predominantly male due to recruitment criteria (Fraser et al., 2013; Fry et al., 2017; Sharp et al., 2020). As with all PRS-based approaches, the underlying biological mechanisms remain uncertain; individual SNPs may tag multiple biological processes via linkage disequilibrium. As noted in our previous study, pathway boundaries are overlapping and imprecise (Harrison et al., 2023). We used summary statistics from Kunkle et al. (2019) a large clinically defined AD GWAS that excludes UK Biobank, thereby maintaining discovery–target independence in both cohorts. Although the more recent Bellenguez et al. (2022) GWAS increases power, it incorporates UK Biobank (including AD-by-proxy), which would reduce independence for the present analyses. Future studies should assess the generalizability of pathway-specific effects using Bellenguez-based scores, and those using UK Biobank should also examine whether pathway-specific PRS show stronger associations in participants with a positive AD-by-proxy phenotype.

A key methodological consideration is that the diffusion MRI pipelines differed substantially between cohorts. The UK Biobank analysis employed TBSS, which, while widely used, is known to have limited spatial specificity and reduced sensitivity to small or curved tracts–particularly those near cerebrospinal fluid or gray matter boundaries (Smith et al., 2006; Bach et al., 2014). For example, the fornix showed no significant associations in the UK Biobank cohort despite its known relevance to AD. It is anatomically narrow, highly curved, and runs adjacent to the ventricles, making it particularly difficult to delineate with TBSS. ALSPAC diffusion data were analyzed with native-space tractography to maximize anatomical specificity in small, curved tracts adjacent to CSF (e.g., fornix), which can be challenging for skeleton-based TBSS approaches. We modeled UKBB scanner site as a covariate, consistent with common practice. Alternative harmonization approaches, such as ComBat and longitudinal ComBat, can further reduce unwanted site/batch variance. Future work should consider harmonized within-cohort pipelines to balance spatial specificity and cross-dataset comparability (Beer et al., 2020).

Finally, interpreting diffusion MRI measures is inherently complex. Both lower FA and higher MD are non-specific and may reflect a range of underlying biological changes, including demyelination, axonal loss, edema, or fiber crossing (Beaulieu, 2002; Jones et al., 2013). As such, caution is warranted when attributing diffusion changes directly to neurodegeneration. Although we focused *a priori* on tracts with strong evidence for early AD vulnerability (parahippocampal and dorsal cingulum, fornix), pathway-specific genetic effects could extend to additional association and prefrontal pathways, particularly in younger adults. Systematic whole-brain or frontally focused extensions will be an important target for future studies, incorporating multimodal neuroimaging and functional genomic annotation, to clarify the molecular and structural pathways linking polygenic risk to brain changes.

This study provides new evidence that polygenic risk for Alzheimer’s disease, stratified according to biological pathway, is associated with differences in white matter microstructure in cognitively healthy older adults. The strongest associations were observed in tracts vulnerable to early AD pathology, such as the parahippocampal cingulum and dorsal cingulum, and surpassed nominal significance threshold after exclusion of the *APOE* locus. In contrast, no robust associations were detected in a younger cohort, despite using harmonized genetic methods and a targeted set of tracts. These findings support the hypothesis that the neuroanatomical effects of AD genetic risk may be developmentally regulated, with minimal impact in early adulthood and greater expression in mid-to-late life. By applying a pathway-specific polygenic approach to large imaging cohorts across the lifespan, this study highlights the value of white matter microstructure as a potential intermediate phenotype for understanding how AD risk unfolds across development and underscores the importance of lifespan and mechanistic perspectives in genetic neuroimaging research.

## Data Availability

Publicly available datasets were analyzed in this study. This data can be found here: The datasets analyzed in this study are accessible upon request from ALSPAC and UK Biobank. Both studies provide comprehensive, searchable data dictionaries and variable search tools on their respective websites to support data discovery (http://www.bristol.ac.uk/alspac/researchers/our-data/, https://www.ukbiobank.ac.uk/).

## Footnotes

1
https://biobank.ctsu.ox.ac.uk/crystal/ukb/docs/genotyping_sample_workflow.pdf

## Supplementary material

Supplementary Figure 1 Associations with the Tau Protein Binding PRS and diffusion metrics in UK Biobank (*n* = 18 172). Pathway-specific polygenic scores were negatively associated with FA in the dorsal and parahippocampal cingulum and positively associated with MD in the same regions. There were no associations with FA or MD in the fornix that withstood multiple comparisons correction. Imaging phenotypes are shown on the *x*-axis, the R^2^ multiplied with the sign of the B-coefficients (positive and negative) are shown on the *y*-axis. Any nominally significant results are labeled with their nominal *P*-value. Each bar represents a version of the PRS, color-coded by the *P*-value threshold used in the training data, shown on the legend. “*P*-value threshold” denotes the SNP inclusion threshold for PRS construction (not a training/validation split). Numerical coefficients, standard errors, confidence intervals, and *p*-values for each model are provided in Supplementary Tables.
