# Supplementary figures and images for: White matter microstructure in mid- to late adulthood is influenced by pathway-stratified polygenic risk for Alzheimer’s disease
Source: Front Neurosci. 2025 Oct 28;19:1638503. doi: 10.3389/fnins.2025.1638503 (PMC12602405; doi:10.3389/fnins.2025.1638503)

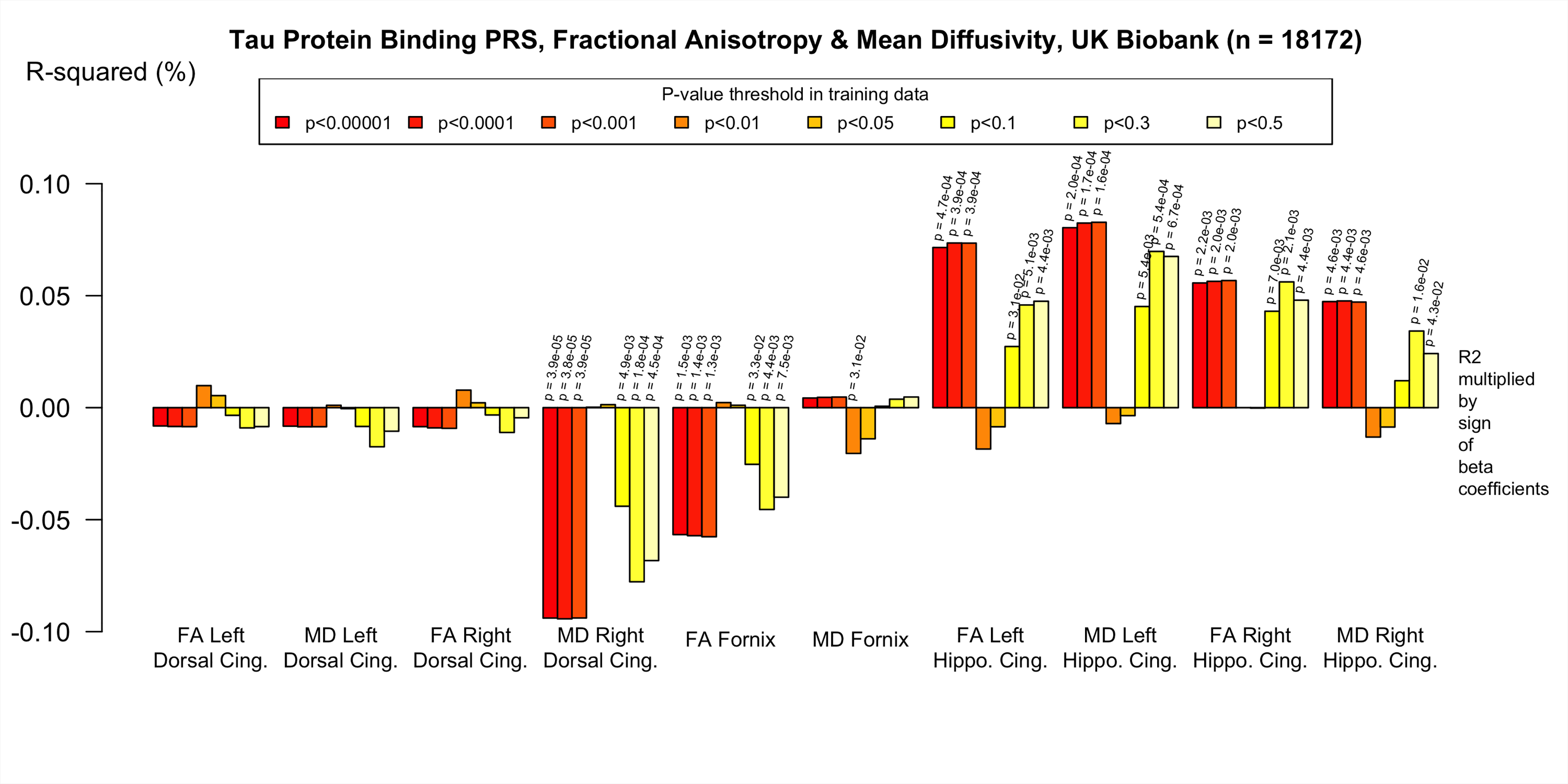

Supplement: Supplementary Figure 1 — Associations with the Tau Protein Binding PRS and diffusion metrics in UK Biobank (n = 18 172). Pathway-specific polygenic scores were negatively associated with FA in the dorsal and parahippocampal cingulum and positively associated with MD in the same regions. There were no associations with FA or MD in the fornix that withstood multiple comparisons correction. Imaging phenotypes are shown on the x-axis, the R2 multiplied with the sign of the B-coefficients (positive and negative) are shown on the y-axis. Any nominally significant results are labeled with their nominal P-value. Each bar represents a version of the PRS, color-coded by the P-value threshold used in the training data, shown on the legend. “P-value threshold” denotes the SNP inclusion threshold for PRS construction (not a training/validation split). Numerical coefficients, standard errors, confidence intervals, and p-values for each model are provided in Supplementary Tables. [file Image_1.tiff]
